# Short Chain *N*-Acyl Homoserine Lactone Production in Tropical Marine *Vibrio sinaloensis* Strain T47

**DOI:** 10.3390/s140712958

**Published:** 2014-07-18

**Authors:** Pui-Wan Tan, Wen-Si Tan, Nina Yusrina Muhamad Yunos, Nur Izzati Mohamad, Tan-Guan-Sheng Adrian, Wai-Fong Yin, Kok-Gan Chan

**Affiliations:** Division of Genetics and Molecular Biology, Institute of Biological Sciences, Faculty of Science, University of Malaya, Kuala Lumpur 50603, Malaysia; E-Mails: acelinetan38@yahoo.com (P.-W.T.); tmarilyn36@gmail.com (W.-S.T.); ninayusrina@hotmail.com (N.Y.M.Y.); zetty_mohamad@yahoo.com (N.I.M.); adrian_tan_1991@yahoo.com (T.-G.-S.A.); yinwaifong@yahoo.com (W.-F.Y.)

**Keywords:** 16S rRNA, biofilm, *Chromobacterium violaceum* CV026, marine seawater-borne bacteria, *N*-acylhomoserine lactone (AHL), *N*-butyryl-L-homoserine lactone (C4-HSL), quorum sensing (QS)

## Abstract

Quorum sensing (QS), acts as one of the gene regulatory systems that allow bacteria to regulate their physiological activities by sensing the population density with synchronization of the signaling molecules that they produce. Here, we report a marine isolate, namely strain T47, and its unique AHL profile. Strain T47 was identified using 16S rRNA sequence analysis confirming that it is a member of *Vibrio* closely clustered to *Vibrio sinaloensis*. The isolated *V. sinaloensis* strain T47 was confirmed to produce *N*-butanoyl-L-homoserine lactone (C4-HSL) by using high resolution liquid chromatography tandem mass spectrometry. *V. sinaloensis* strain T47 also formed biofilms and its biofilm formation could be affected by anti-QS compound (cathechin) suggesting this is a QS-regulated trait in *V. sinaloensis* strain T47. To our knowledge, this is the first documentation of AHL and biofilm production in *V. sinaloensis* strain T47.

## 1. Introduction

Bacteria are able to communicate in a cell-to-cell manner where they rely on the production and responses to extracellular signaling molecules known as autoinducers to monitor gene expression that is cell density dependent [1,2]. This phenomenon is termed “quorum sensing (QS)” as this system enables bacteria to act in unison by synchronizing their gene expression [3,4]. The most well documented autoinducers are the *N*-acylhomoserine lactones (AHLs) which are used by most Gram-negative bacteria that typically involves the LuxI/LuxR complex in order to regulate the communication [5]. AHLs are synthesized by LuxI homologs (AHL synthases) where AHL will bind to the LuxR receptor to form the AHL-LuxR complex which regulates QS-dependent gene expression [6,7]. The AHL/LuxR complex will then be in an active form that can regulate the QS-dependent genes. Hence, AHLs produced by various microorganisms can differ with respect to the length with various substitutions at the carbon-3 position with the presence or absence of unsaturation within the acyl side chains [8]. Generally, the type of AHL is specific to the target receptor, and hence QS is AHL specific and dependent.

QS microorganisms can be ubiquitous, and they thrive in different habitats. The marine environment could be a habitat for diverse and dense bacterial communities including QS microorganisms. *Vibrio* species usually represent the major microorganisms in the marine environment where they could cause severe infections in many marine organisms such as fish and prawns and the humans that consume them. Most of the pathogenic *Vibrio* studied are normally opportunistic and can pose a threat to aquaculture farming [9,10]. Del Carmen and colleagues showed that infectious diseases, especially those caused by bacterial and viral pathogens, lead to serious losses in shrimp farming where they are hosted in the gut and hepatopancreas of stressed shrimps [10]. This serious disease is caused by *Vibrio sinaloensis* [10]. In this study, we reported the AHL production by this marine isolate.

## 2. Experimental Section

### 2.1. Marine Water Sample Collection and Isolation of Bacterial Strain

The sampling site chosen for this study was Morib Beach (GPS coordinates: N02°45.023′ E101°26.623′) which is located in Selangor, Malaysia in 2013. A water sample was collected in a sterile plastic bottle (50 mL) along the beach coastal area at a depth of 20 cm below water surface. It was then kept at 4 °C before until further analysis [11]. A serial dilution of marine water sample was carried out with sterile saline (0.9% w/v NaCl). Bacterial culture was spread onto Luria Bertani (LB) agar (in grams per 1 L: tryptone, 10; yeast extract, 5; NaCl, 30; Bacto agar, 15) and incubated overnight (24 h) at 28 °C. Colonies of different morphologies were isolated and pure colony was obtained by repeated streaking on LB agar.

### 2.2. Bacteria Strains, Culture Conditions and Bacterial Biosensor Assay

Colonies obtained were screened by using a AHL biosensor, namely *Chromobacterium violaceum* CV026. Strain T47 activated *C. violaceum* CV026 [12] and was selected for further studies. This isolate was routinely cultured on LB medium. *Erwinia carotovora* GS 101 producing AHL molecules (*N*-3-oxohexanoyl-homoserine) was used as positive control for AHL production screening involving *C. violaceum* CV026. To further verify the AHL production by strain T47, *Escherichia coli* [pSB 401] was used as another *lux*-based biosensor that will produce bioluminescence in the presence of short chain AHLs [13]. The positive and negative controls for the screening were *Erwinia carotovora* GS101 and *E. carotovora* PNP22, respectively. *C. violaceum* CV026, *E. carotovora* GS101 and *E. carotovora* PNP22 were routinely maintained on LB agar.

### 2.3. Preliminary Screening of AHLs with Bacterial Biosensors

Isolate T47 was screened for AHL production by cross streaking the bacterial culture with *C. violaceum* CV026 on an LB agar plate and incubating overnight (24 h) at 28 °C. After incubation, purple violacein pigmentation by *C. violaceum* CV026 indicates the production of AHL [12]. For detection of *lux*-based biosensor *E. coli* [pSB401], a photon camera with 60 s of exposure time was used to observe the induced bioluminescence after 24 h incubation at 28 °C. Bioluminescence indicates AHL detection by *E. coli* [pSB401].

### 2.4. Bacteria Identification

Amplification of bacterial 16S rRNA genes with polymerase chain reaction (PCR) was carried out as previously described [14]. For PCR amplification, we used PCR mix obtained from Promega (Promage Kit, Madison, WI, USA). Genomic DNA extraction was done using MasterPureTM DNA Purification Kit (Epicentre Inc., Madison, WI, USA). PCR amplification and purification process was conducted as described previously [14]. PCR product sequence alignment was done using GenBank BLASTN program followed by phylogenetic analysis using the Molecular Evolutionary Genetic Analysis version 6.0 [15,16].

### 2.5. AHLs Extraction

Strain T47 was cultured in LB broth buffered with 50 mM of 3-(*N*-morpholino)propanesulfonic acid (MOPS) (pH 5.5) and incubated with shaking (200 rpm, 28 °C, 18 h). Overnight culture supernatant was extracted twice with addition of equal volume of acidified (0.1% v/v glacial acetic acid) ethyl acetate and mixed vigorously [7]. Organic solvent was collected and dried in the fume hood and the extract was resuspended with 1 mL of acidified ethyl acetate and dried again. After that, 200 μL of acetonitrile (HPLC grade) was added to dissolve the AHL extracts for further analysis.

### 2.6. AHL Profiling by Mass Spectrometry (MS)

An Agilent RRLC 1200 system was utilized as the liquid chromatography (LC) delivery system with the use of Agilent ZORBAX Rapid Resolution HT column and the MS parameters were fixed as reported previously [17]. The high resolution electrospray ionization mass spectrometry (ESI-MS) was performed with an Agilent 6,500 Q-TOF LC/MS system operated in ESI-positive mode. The *m/z* value range to detect the precursor ions was set at *m/z* 150–400. The precursor ion scan mode targeting at the production ion with *m/z* 102. Analysis of MS spectra generated and AHL profile was performed as described previously [17].

### 2.7. Biofilm Assay

Biofilm assay was performed as described previously [18]. Overnight culture of strain T47 was diluted with sterile LB medium, and adjusted to OD_600_ of 0.1. Subsequently, diluted culture (50 μL) was dispensed into a microtitre well containing 930 μL of sterile LB medium supplemented with 1 mg/mL of anti-QS compound (catechin) [18]. Strain T47 cultures treated with and without DMSO were used as negative and positive controls, respectively. Strain T47 was incubated statically for 2 days at 28 °C. Unattached planktonic cells were removed by gently washing with sterile distilled water followed by air-dried for 15 min aseptically. To stain the biofilm, the wells were filled with 200 μL of 0.1% (w/v) crystal violet for 30 min, washed twice with sterile distilled water, followed by addition of 200 μL of 95% (v/v) ethanol. Amount of biofilm formed was measured by transferring 100 μL of this ethanol solution to a new microtitre plate and reading the absorbance at OD_590_ with a microplate reader. These biofilm assays were repeated twice.

## 3. Results and Discussion

### 3.1. Strains Isolation and Preliminary Screening of AHL

The aim of this study was to isolate AHL-producing bacteria from a marine seawater sample and characterize its AHL profile. The sampling site chosen for this study is Morib Beach which is in an area near a fishing village. The temperature and pH of the water sample collected was recorded at 27 °C and pH 8.0, respectively. The marine water sample was collected near the coastal line to determine whether pathogenic bacteria that could infect either humans or affect fishing activities can be detected.

**Figure 1.**
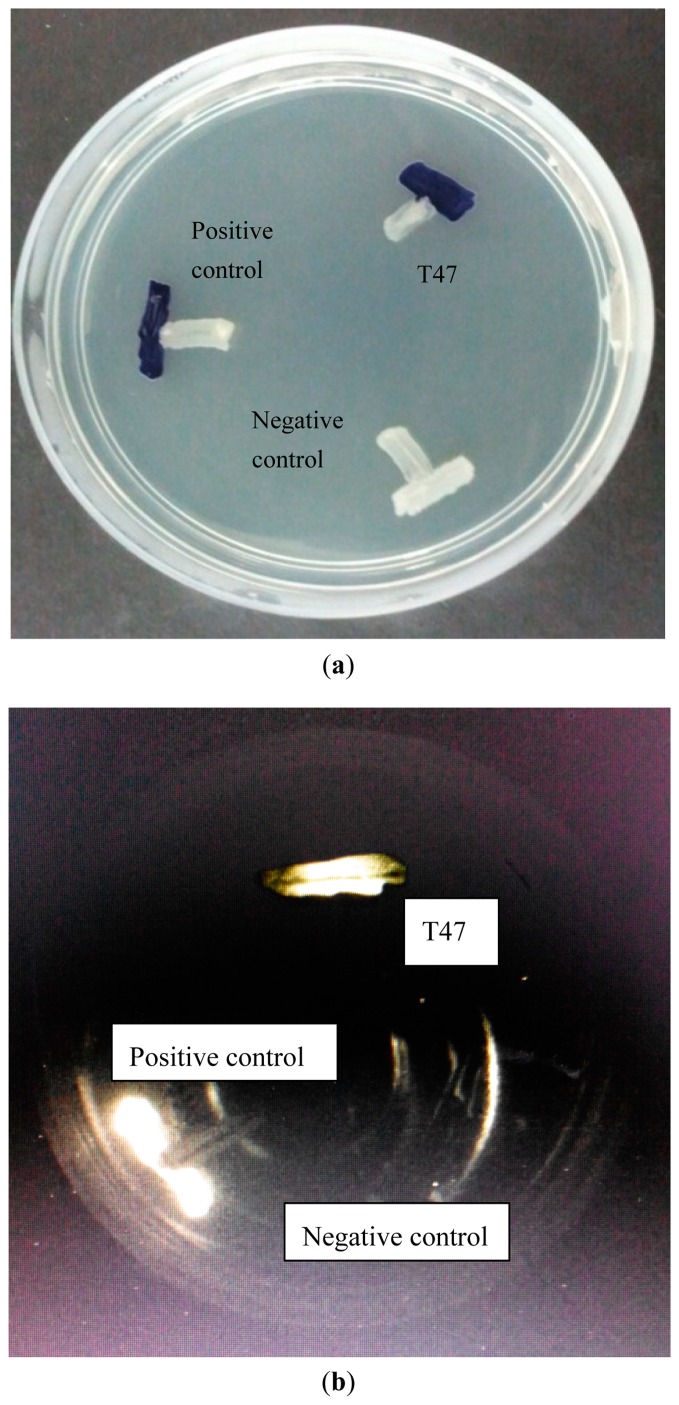
(**a**) AHL screening of strain T47 with *C. violaceum* CV026 and (**b**) with *E. coli* pSB401. *E. carotovora* PNP22 (negative control) devoid of QS activity was included and *E. carotovora* GS101 (positive control) that can activate *C. violaceum* CV026 and *E. coli* pSb401 was included.

The availability of the AHL biosensors has led to the discovery of many QS bacteria [19]. AHL biosensors are mutants with defective LuxI AHL synthase, which practically rely on the LuxR protein to display specific binding towards the cognate AHL that is able to activate the transcription of the reporter gene [20]. The biosensor *C. violaceum* CV026 responds to the AHLs with C4 to C8 acyl chain length that will induce a purple violacein pigmentation [12]. It is a preferable biosensor for AHL preliminary screening due to the rapidness and accuracy in its AHL detection. Hence, we used *C. violaceum* CV026 for the preliminary screening of AHLs produced by strain T47 (Figure 1). The induction of purple violacein in the *C. violaceum* CV026 biosensor indicated that the isolated strain T47 produced detectable short chain AHLs. In addition to this, T47 also induced bioluminescence in *E. coli* [pSB401]. Both biosensor tests with *C. violaceum* CV026 and *E. coli* [pSB401] indicated that strain T47 produced short chain AHLs. The strain was next subjected to AHL profiling and molecular identification.

### 3.2. Molecular Identification of Bacterial Strain

**Figure 2.**
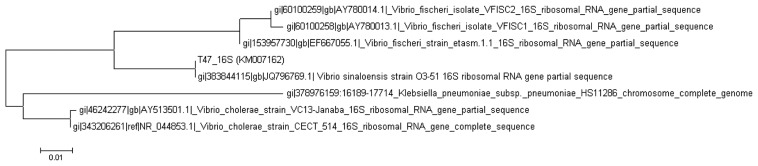
Phylogenetic analysis of strain T47. Phylogenetic tree reconstructed using the Maximum likelihood algorithm showing phylogenetic relationships of strain T47 to members of the other species of the genus *Vibrio*. Bar, 1 substitution per 100 nucleotides positions. Gene accession numbers are indicated.

The identity of the strain T47 was confirmed by analysis of its 16S rRNA gene nucleotide sequences showing that it clustered closely within the *Vibrio* genus, where the strain shared 99% similarity in the BLAST search. Based on the phylogenetic tree constructed (Figure 2), strain T47 was identified as *Vibrio sinaloensis*, a marine opportunistic bacterium.

### 3.3. Analytical Identification of AHL Molecules

*Vibrio* genus is a causative agent for food-borne diseases and in many countries, it has infected patients who consumed undercooked seafood and mortality has been reported [21]. For example, the species *V. alginolyticus* was isolated in 1997 from patients during an outbreak of acute enteric illness in Vladivostok, Russia [22]. In addition to this, foodborne illness caused by *V. alginolyticus* has been identified in 96 cases after the patients consumed brine shrimp in Chifeng Hongshan, China in 2004 [23]. Although we have reported very detailed QS systems in some of the members of *Vibrio*, this is almost entirely limited to a few model *Vibrio* but little is known about QS in *V. sinaloensis* strain T47. Moreover, the pathogenicity of *V. sinaloensis* is still largely unknown. Interestingly, our work showed that this bacterium produced AHLs which are the vital QS signalling molecules in proteobacteria. It is believed that this AHL is responsible in regulating certain QS physiological activities of *V. sinaloensis* [24]. Thus, the availability of detecting AHL profile of *Vibrio* sp. represents the first step in understanding this bacterium's QS gene regulation.

**Figure 3.**
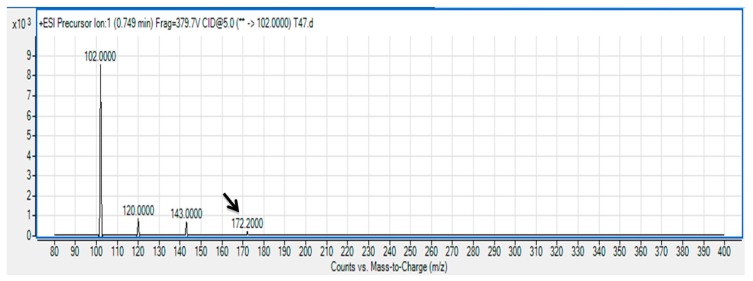
Mass spectrometry analysis of spent supernatants extract of *Vibrio sinaloensis* strain T47. Mass spectrum of C4-HSL (*m/z* 172.2000) (arrow).

The spent culture supernatant of *V. sinaloensis* strain T47 was analyzed using mass spectrometry in order to determine the exact AHL produced by *V. sinaloensis* strain T47. The presence of *N*-butanoyl-L-homoserine lactone (C4-HSL) [25,26] with retention time of 0.749 min was identified and confirmed (Figure 3). To our knowledge, this is the first documentation on AHL profiling of *V. sinaloensis* where it produces AHL molecule as part of its QS system. There are three major QS systems in the Vibrionaceae family namely *luxI*, *hdts* and *luxM*. The majority of AHL synthases identified show either formation of 3-hydroxy-HSLs or 3-oxo-HSLs along with or without unsubstituted AHLs of the same or similar acyl chain lengths [26]. Purohit *et al.* [24] reported that they have identified 3-hydroxy-C8-HSL in *Aliivibrio fischeri* ES114, which is produced at a very low concentration. Hence it will not be sufficient to predict the AHL-producing QS system simply by analyzing the AHLs. This result enables us to take a deeper approach into studying the mechanism of QS of *V. sinaloensis*. Besides that, our group also expanding this research to the whole genome sequence to gain more insights on the *luxI* and *luxR* genes in this strain.

A battery of physiological activities such as biofilm formation, virulence factors and motility can be regulated by QS [1–3] and hence this work provides the basis to illustrate the significance of research on AHL-producing bacteria present in environmental samples. *V. sinaloensis* causes serious losses in shrimp and fish farming. Our work provides valuable input to explore anti-QS [27,28] as a tool to attenuate marine QS pathogens which can form the basis of novel treatments in marine aquaculture.

### 3.4. Biofilm formation

**Figure 4.**
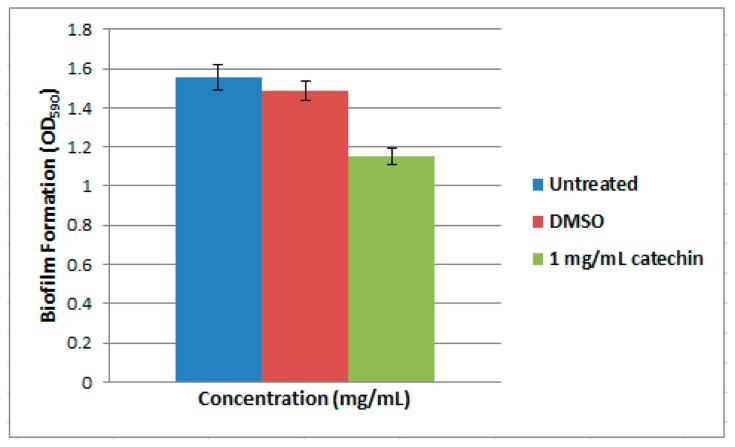
Qualitative analyses of biofilm formation and inhibition by anti-QS compound, catechin. Bars: Standard errors of the mean.

Numerous studies have investigated biofilm formation in *Vibrio* species such as *V. cholera*, *V. parahaemolyticus*, *V. vulnificus* and *V. fischeri* [29]. Biofilms formed at solid-liquid interfaces have been analyzed under static or flow conditions [29]. In our study, we analyzed biofilm formation of strain T47 under static culture conditions. Bacteria colonization is initiated, followed by surface attachment, resulting in the formation of a mature biofilm. For example, *V. vulnificus* and *V. parahaemolyticus* which are found on surfaces of plankton and colonize shellfish [29]. Strain T47 has been shown to be able to form biofilm (Figure 4). Catechin, an anti-QS compound [30], was used in this study to inhibit the biofilm formation in strain T47. The biofilm formation of strain T47 was reduced in a dose-dependent manner (Figure 4). Since *Vibrio* species use QS to control numerous traits, including bioluminescence, virulence and biofilm formation this work illustrated the significance of expanding the research on AHL-producing bacteria present in environmental samples. Hence, in this work we suggest that sea water may serve as a potential reservoir for QS pathogens that should be given appropriate attention.

## 4. Conclusions

Here we report the AHL profile of *V. sinaloensis* T47 isolated from tropical marine seawater which confirmed that this isolate produced a short chain AHL *viz.* C4-HSL. This is the first documentation of *Vibrio sinaloensis* producing this AHL.
